# Evidence of Validity for a Newly Developed Digital Cognitive Test Battery

**DOI:** 10.3389/fpsyg.2020.00770

**Published:** 2020-04-24

**Authors:** Stefan Vermeent, Ron Dotsch, Ben Schmand, Laura Klaming, Justin B. Miller, Gijs van Elswijk

**Affiliations:** ^1^Digital Cognitive Diagnostics, Philips Healthcare, Eindhoven, Netherlands; ^2^Department of Brain, Behavior and Cognition, Philips Research, Eindhoven, Netherlands; ^3^Cleveland Clinic Lou Ruvo Center for Brain Health, Las Vegas, NV, United States

**Keywords:** structural validity, digital testing, digital cognitive test battery, confirmatory factor analysis, Cattell-Horn-Carroll model

## Abstract

Clinical practice still relies heavily on traditional paper-and-pencil testing to assess a patient’s cognitive functions. Digital technology has the potential to be an efficient and powerful alternative, but for many of the existing digital tests and test batteries the psychometric properties have not been properly established. We validated a newly developed digital test battery consisting of digitized versions of conventional neuropsychological tests. Two confirmatory factor analysis models were specified: a model based on traditional neuropsychological theory and expert consensus and one based on the Cattell-Horn-Carroll (CHC) taxonomy. For both models, the outcome measures of the digital tests loaded on the cognitive domains in the same way as established in the neuropsychological literature. Interestingly, no clear distinction could be made between the CHC model and traditional neuropsychological model in terms of model fit. Taken together, these findings provide preliminary evidence for the structural validity of the digital cognitive test battery.

## Introduction

Neuropsychological tests are an invaluable part of the clinician’s assessment toolbox when there is reason to suspect an impairment in someone’s cognitive functioning. Most of the standard neuropsychological tests have a long history in the field and have traditionally been administered in paper-and-pencil form. However, assessing cognitive functioning using paper-and-pencil tests has major limitations: it is labor-intensive on the side of the clinician in terms of administration and scoring, provides little flexibility in stimulus use or updating of tests, and is severely limited in the type of outcome measures that can be extracted (Miller and Barr, 2017; Klaming and Vlaskamp, 2018). Digital cognitive testing effectively addresses some of these issues (Bauer et al., 2012; Riordan et al., 2013; Zygouris and Tsolaki, 2015; Feenstra et al., 2017; Galindo-Aldana et al., 2018; Germine et al., 2019; Kessels, 2019).

Despite their many advantages, the adoption of digital cognitive tests is not straightforward. Most importantly, it has often been argued that the psychometric properties of many digital tests have not been properly established (e.g., Schlegel and Gilliland, 2007; Wild et al., 2008; Bauer et al., 2012). This is problematic, because it cannot be assumed that paper and digital versions of the same test will measure the same underlying cognitive domains (Bauer et al., 2012; American Educational Research Association, 2014). Currently, the evidence for agreement between paper and digital tests is mixed at best, with some studies showing no performance differences between paper-and-pencil and digital tests (Williams and McCord, 2006; Parsey and Schmitter-Edgecombe, 2013) while others demonstrate substantial differences (Williams and Noyes, 2007; Riordan et al., 2013; Feenstra et al., 2017; Carpenter and Alloway, 2018).

In this article, we present a newly developed digital cognitive test battery (DCTB). Several integrated digital testing platforms already exist with intermediate levels of automation (e.g., Pearson’s Q-Interactive, which has automatic scoring but requires the clinician to provide the instructions and control visual stimuli) that have well-established psychometric properties. Others, such as the National Institutes of Health (NIH) Toolbox (Weintraub et al., 2013), consist of tests that were developed exclusively for the platform and are only loosely based on existing paper-and-pencil tests. The aim of the current DCTB is to provide a set of digital tests that are based on conventional paper-and-pencil tests and that have a high level of automation in stimulus presentation, scoring, and interpretation. Since uncertainties concerning validity prevent large-scale adoption of digital technology in clinical practice (Schmand, 2019), a first important step is to collect evidence of validity for the DCTB in terms of the cognitive domains that it measures.

Clinicians generally rely on interpreting test performance in terms of underlying cognitive domains. However, there is an ongoing discussion on the exact domains that should be distinguished and which cognitive tests contribute to which cognitive domain (Larrabee, 2014). This issue is exacerbated by the fact that the majority of tests are sensitive to multiple cognitive abilities. For example, the Trail Making Test (TMT) could be construed as a measurement of executive functioning, attention, and sensory-motor functioning (Rabin et al., 2016). Confirmatory factor analysis (CFA) can assist in quantifying these relationships and converging on a plausible factor structure and is therefore a widely used technique to gather evidence of validity (Jackson et al., 2009). Factor models in the neuropsychological literature are generally in agreement with traditional theoretical classifications (e.g., Strauss et al., 2006; Lezak et al., 2012), distinguishing domains such as executive functioning, memory, attention, language, and visual-spatial processing (e.g., Siedlecki et al., 2008; Dowling et al., 2010; Hayden et al., 2011; Park et al., 2012).

A competing model that has gained increasing attention in recent years in the neuropsychological field is the Cattell-Horn-Carroll (CHC) model (e.g., Jewsbury et al., 2017; Agelink van Rentergem et al., 2020). The CHC model was originally developed in the intelligence literature as a synthesis of Carroll’s three-stratum model and the Cattell-Horn model and has a hierarchical structure (for an extensive review of the history of the CHC model, see McGrew, 2009). Test scores are clustered under *narrow abilities* (e.g., retrieval fluency, learning efficiency), which are in turn clustered under *broad abilities* (e.g., long-term storage and retrieval). At the very top of the hierarchy, a general factor “g” is usually specified to account for covariances between the broad abilities (Schneider and McGrew, 2018). However, the nature and even existence of g remains a point of debate (Wasserman, 2019). The CHC model features cognitive domains (i.e., broad abilities) that differ from the ones that are commonly used in neuropsychological research and practice. Examples of such domains are fluid reasoning and crystallized abilities. The most pronounced difference between the CHC model and more traditional neuropsychological models is the absence of a distinct executive functioning factor in the former (Jewsbury et al., 2017). Instead, tests that are traditionally linked to executive functioning are distributed across multiple domains in the CHC model, such as processing speed, fluid reasoning, and retrieval fluency (Jewsbury et al., 2017; Agelink van Rentergem et al., 2020). Floyd et al. (2010) argued that executive functioning measures are contaminated by general intelligence to the point that executive functioning as a distinct concept no longer has relevant explanatory power. Thus, the question whether the construct of executive functioning is needed and can be seen as a unitary entity is still a point of debate (e.g., Miyake et al., 2000; Friedman and Miyake, 2017; Karr et al., 2018).

Here, we present evidence for the validity of our newly developed DCTB, consisting of digital versions of 11 of the most commonly used paper-and-pencil tests in neuropsychological practice. Validity evidence based on the internal structure of the DCTB (American Educational Research Association, 2014), was tested by fitting the test scores to a neuropsychological consensus model and to the CHC model. Based on recent neuropsychological literature (Jewsbury et al., 2017; Agelink van Rentergem et al., 2020), we expected that the CHC model would perform better in terms of model fit than the neuropsychological consensus model.

## Materials and Methods

### Participants

A total of 265 healthy participants were recruited in two samples. Of these, 209 healthy middle-aged and elderly participants were recruited by a recruitment agency through their database. Participants were eligible for participation if they were 50 years or older. An additional sample of 56 healthy participants were recruited in collaboration with the University Medical Centre Utrecht (UMCU) as a control group in their study on a diverse patient group. These participants were recruited among colleagues, (sports) associations, and the social network of (former) outpatients of the UMCU. In this sample, individuals were considered eligible for participation if they were between 18 and 80 years old. Although the inclusion of this sample skewed the age distribution, with most participants within 50 and 80 years old, it was deemed informative to have a broad age coverage. In addition, the final sample size of 265 was more adequate in the context of CFA. It should be emphasized that both samples were convenience samples in the sense that their data were not collected with the primary aim of establishing validity evidence but were for the most part already collected.

In both samples, participants were included if they were fluent in Dutch and had (corrected to) normal eyesight and hearing. Participants were excluded if they reported to have severe communication, motor, neurological or psychiatric disorders; were unable to use a tablet to perform the digital tests; consumed more than three glasses of alcohol per day or were recreational drug users; used any psychopharmacological drugs; or had performed a traditional neuropsychological assessment in the 6 months prior to the study to rule out potential re-test effects. We screened for potential cognitive impairment using the paper-and-pencil version of the Mini Mental State Examination second edition (MMSE-2; Folstein et al., 2010). Participants were excluded if their MMSE-2 score was below 24.

Of the full sample, four participants (1.5%) were excluded due to medication use, five (1.9%) due to technical difficulties, four due to colorblindness (1.5%), and four (1.5%) due to being unable to finish the full test battery, which precluded the use of their data in the CFA analyses. Finally, two participants (0.8%) were excluded because their MMSE scores were below 24, and seven participants (2.6%) were excluded because their scores on one or more of the outcome measures included in the models were extreme outliers (>3.2 SD). After these exclusions, 239 participants remained for further analyses. An overview of their demographic information is presented in Table 1.

**TABLE 1:** Participant characteristics.

***N* = 239**	**M ± SD**	**Range**
Gender (% female)	40.6	
Age (years)	61 ± 12.5	21–81
Education level^*a*^	5.7 ± 1.0	0–6
MMSE-2	28.9 ± 1.2	25–30

*^*a*^ISCED (UNESCO, 1997/2006): 0 = pre-primary education; 1 = primary education or first stage of basic education; 2 = lower secondary or second stage of basic education; 3 = (upper) secondary education; 4 = post-secondary non-tertiary education; 5 = first stage of tertiary education; 6 = second stage of tertiary education.*

### Materials

#### Digital Neuropsychological Tests

The DCTB was composed of tests that are used internationally in multiple languages, have well-established psychometric properties in their paper-and-pencil version, and together cover a broad range of cognitive domains. Approximately half of the tests were scored using automated algorithms and the other half were scored manually (see Table 2 for a description of these tests and how they deviate from their paper-and-pencil versions).

**TABLE 2:** Descriptions of the tests in the digital cognitive test battery and the differences from pen-and-paper versions.

**Test**		**Description**	**Difference from pen-and-paper version**
1.	Trail-Making Test A^*a*^	Participants connect circles labeled 1 to 25 as fast as possible	Drawing is done on an iPad; Automated scoring
2.	Trail-Making Test B^*a*^	Participant alternately connect circles containing letters (A–H) and numbers (1–13) as fast as possible	Drawing is done on an iPad; Automated scoring
3.	Digit span forward	Participants verbally repeat back digit strings of increasing length	Automated verbal stimulus presentation; Automated scoring
4.	Digit span backward	Participants verbally repeat back digit strings of increasing length in reverse order	Automated verbal stimulus presentation; Automated scoring
5.	COWAT	Over three trials, participants name as many (Dutch) words starting with a “D”, “A”, and “T”.	-
6.	CFT, category ‘animals’	Participants name as many words that fall in the category ‘animals’	−
7.	Stroop color-naming^*a*^	Participants name the color in which congruent color-words are presented as fast as possible	Digital stimulus presentation
8.	Stroop interference^*a*^	Participants name the color in which incongruent color-words are presented as fast as possible	Digital stimulus presentation
9.	RAVLT learning trials	Over five learning trials, participants verbally repeat back as many words as possible from a fixed word list containing 15 items	Automated verbal stimulus presentation
10.	RAVLT delayed recall	10–20 min after completion of the learning trials, participants verbally repeat back as many words as possible from the original word list	Automated verbal stimulus presentation
11.	ROCFT copy	Participants are presented with a complex figure and are asked to copy it	Drawing is done on an iPad
12.	ROCFT immediate recall	Participants draw the complex figure from memory	Drawing is done on an iPad
13.	Star-Cancellation Test^*a*^	Participants cross out all the star stimuli in a field containing distractors	Digital stimulus presentation; Drawing is done on an iPad; Automated scoring
14.	O-Cancellation Test^*a*^	Participants cross out all the O stimuli in a field containing distractors	Digital stimulus presentation; Drawing is done on an iPad; Automated scoring
15.	Clock Drawing Test	Participants draw a clock from memory with the hands indicating 10 past 11	Drawing is done on an iPad
16.	Card Sorting Test	Participants match stimulus cards containing various shapes in different numbers and colors according to implicit (and changing) sorting rules	Digital stimulus presentation; Automated scoring

### Procedure

Both studies received approval from the institutional review board. The study by the UMCU was waived by the Medical Ethical Committee. Participants gave written informed consent at the beginning of the study. All tests were administered digitally using a software prototype of the DCTB as it is envisioned for future public release. Participants completed the DCTB on the Apple iPad Pro 12.9-inch (2nd generation) with a screen resolution of 2732 × 2048 that was supplied by the digital platform developers. Tests that required drawing made use of the Apple Pencil for the iPad Pro. Audio recordings used for scoring were made using the internal microphone for tests that required a verbal response.

For the participants recruited through the recruitment agency, testing took place during a single visit to our research facility. Half of these participants were tested by a trained experimenter, and the other half by a neuropsychologist. All data were scored by neuropsychologists. The participants recruited by the UMCU were tested at home or at the UMCU. The decision to test some of the participants of the UMCU study at home was especially taken to facilitate patients (not included in this study) who did not have appointments anymore at the UMCU, but this was also extended to the healthy participants (who were mostly relatives or friends of the patients). In total, 34 participants in the UMCU study were tested at the UMCU and 15 were tested at home. We tested for a potential influence of location on performance through independent-sample *t*-tests but did not find significant differences on any of the outcome measures described in this study (all *p*s > 0.29). These data were collected and scored by four neuropsychology students. The total assessment duration was 1.5 h. All participants entered the study voluntarily and received a compensation for participation and travel expenses upon completing the assessment. Participants did not receive feedback about their performance on the cognitive tests and the results were not used for clinical purposes.

In both studies, the experimenters made sure that the tests were administered with normal room lighting, that is, not directly next to the window with direct sunlight or directly under a lamp to prevent reflections on the iPad. The brightness and volume of the iPad were set to their maximum values. All participants first received a paper-and-pencil version of the MMSE-2, after which their demographic information was entered into the iPad. Next, the DCTB was presented in the following order: Rey Auditory Verbal Learning Test (RAVLT) learning trials, Trail Making Test (TMT), O-Cancellation Test (OCT), Clock Drawing Test (CDT), Star Cancellation-Test (SCT), RAVLT delayed recall, Rey-Osterrieth Complex Figure Test (ROCFT) copy, Controlled Oral Word Association Test (COWAT), ROCFT immediate recall, Digit Span (DS) forward and backward, Category Fluency Test (CFT), Stroop color-word interference Test, ROCFT delayed recall, and a Card Sorting Test (CST). The decision to fix the test order was mainly dictated by the RAVLT delayed recall and ROCFT immediate recall, which were required to be administered after a relatively stable interval. Fixing the test order may have introduced a confound, for example, because participants may have been more systematically fatigued on the later tests. However, since full randomization was not possible without introducing substantial differences in the delays between learning and recall trials, we preferred one fixed order over several pseudo-random orders.

The CDT and CST were excluded from all analyses. Collecting data for the CST was stopped after 104 participants due to time constraints (the CST was one of the longest tests in the DCTB) and had a strong ceiling effect. In addition, commonly used outcome measures of the CST (i.e., total correct, percentage of perseverative errors, and percentage of conceptual level responses) performed poorly in a factor structure with the other tests, which is in line with earlier studies that highlight the lack of validity of traditional outcome measures of the CST (Strauss et al., 2006; Nyhus and Barceló, 2009). Data for the CDT were collected for all participants but not included in the analyses because of a ceiling effect (using a three-point scoring method; Goodglass et al., 2001).

### Evidence for Structural Validity

We used CFA with maximum likelihood estimation to assess validity of the DCTB. Analyses were performed using the *lavaan* package (version 0.6-1; Rosseel, 2012) in the R environment (R Core Team, 2018). The variances of the latent factors were fixed to 1 in the model definition and the loadings were scaled accordingly by the coefficients in the estimation procedure (the variance of single-indicator factors was fixed to 1 minus the reliability of the indicator, see section “Neuropsychological Consensus Model”). Therefore, the covariances between latent factors can be interpreted as correlations. Outcome measures from the same test were allowed to covary, except when: (1) they were the only indicators loading on a factor, in which case their covariance is captured in the latent factor, and (2) when one of the outcome measures was the only indicator of a factor. In the latter case, the latent factor accounts for all the variance in the outcome measure, so there is no error variance left for an additional covariance parameter. Model fit was assessed through several fit indices: Chi square, which compares the model-implied covariance matrix with the sample covariance matrix, and the approximate fit indices Standardized Root Mean Square Residual (SRMR), Comparative Fit Index (CFI), Tucker-Lewis index (TLI) and the Root Mean Square Error of Approximation (RMSEA), which offer a continuous measure of model fit (Kline, 2011). Interpretation of these quantitative fit indices was based on the recommendations made by Schermelleh-Engel et al. (2003): good fit to the data was qualified through a non-significant chi square test (*p* > 0.05), SRMR ≤ 0.05 (acceptable fit ≤ 0.10), RMSEA ≤ 0.05 (acceptable fit ≤ 0.08), and both CFI and TLI ≥ 0.97 (acceptable fit ≥ 0.95). Following Hu and Bentler (1999), we place most reliance on the combination of SRMR and RMSEA. Comparisons of nested models were additionally based on chi square difference tests and the Akaike information criterion (AIC) with lower values indicating better fit.

In the case of non-nested models, a comparison through a chi square difference test is not possible. Instead, for such comparisons we used the Vuong test for non-nested models (Vuong, 1989; Merkle et al., 2016). The Vuong test provides a test of the models’ distinguishability in the population of interest. If they are distinguishable, a further statistical comparison of model fit and the AIC difference is possible. These tests were implemented using the nonnest2 package (Merkle and You, 2018) in R. Any changes made to the model due to non-convergence or based on data-driven modification indices were made with caution and explicitly reported.

Most outcome measures of neuropsychological tests are influenced by demographic factors such as age and education level, which can significantly bias the factor structure if they are unaccounted for Agelink van Rentergem et al. (2020). Therefore, the influences of age, highest completed education, and sex on the raw test scores were assessed through separate linear regression models and, if significant at an alpha-level of 0.10, were partialed out using regression-based norming techniques (Testa et al., 2009). Because of this correction, test scores that entered the model followed a z-score distribution. Education was operationalized by the International Standard Classification of Education (ISCED; scale 0–6; UNESCO, 1997/2006). The ISCED provides a good measure of the person’s intelligence level and facilitates international comparisons.

#### Neuropsychological Consensus Model

The mappings of the neuropsychological consensus model were based on reported classifications in the neuropsychological literature (Strauss et al., 2006; Lezak et al., 2012; Larrabee, 2015), previous CFA studies (Siedlecki et al., 2008; Dowling et al., 2010; Hayden et al., 2011; Park et al., 2012), research on common clinical practice (Rabin et al., 2016), and expert opinions of 20 experienced clinical neuropsychologists (Hoogland et al., 2017). Consequently, the neuropsychological consensus model was guided by theory, clinical practice and expert opinion. It is important to note that there is no single generally accepted classification of cognitive domains, with some domain specifications being more controversial than others (Larrabee, 2015). In addition, not all domains as reported by the sources listed above were included in our neuropsychological consensus model, mostly because the DCTB tested here does not contain tests for certain cognitive domains (e.g., language, motor function).

**FIGURE 1:**
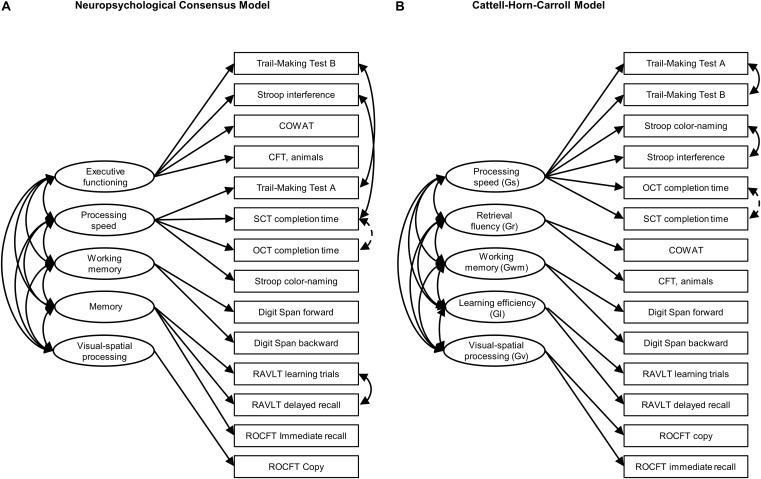
Graphic overview of **(A)** the neuropsychological consensus model and **(B)** the CHC model. Single-headed arrows represent factor loadings, double-headed arrows represent covariances. Covariances printed with solid lines were pre-specified. Covariances printed with dashed lines were added after inspecting modification indices. COWAT, Controlled Oral Word Association Test; CFT, Category Fluency Test; OCT, O-Cancellation Test; SCT, Star-Cancellation Test; RAVLT, Rey Auditory Verbal Learning Test; ROCFT, Rey-Osterrieth Complex Figure Test.

The specification of this model is depicted in Figure 1A. It consists of five cognitive domains: executive functioning, working memory, memory, processing speed, and visual-spatial processing. Executive functioning is broadly specified, containing tests that measure inhibition and task-switching (Friedman and Miyake, 2017; Karr et al., 2018), as well as response monitoring (Lezak et al., 2012). We decided to specify working memory as a separate factor, even though it is often considered a component of executive functioning (e.g., Strauss et al., 2006; Lezak et al., 2012). Clinicians often assess working memory in isolation and recent studies supported models including a separate working memory factor (Dowling et al., 2010; Hayden et al., 2011; Park et al., 2012).

Because Visual-spatial processing constitutes a single-indicator domain, we fixed its variance to 1 minus the reliability of ROCFT Copy. This reliability estimate was obtained from Strauss et al. (2006), who report on studies finding estimates as low as 0.18 and ranging between 0.57 and 0.77. For the final model, we decided on a reliability estimate of 0.60. However, conclusions reported below are identical using other estimates (e.g., 0.18, 0.57, 0.77, or 1). It should be noted that these reliability estimates are derived from the paper-and-pencil literature and that it is currently unknown whether the reliability of the digital ROCFT is comparable.

#### CHC Model

For the construction of the CHC model, we followed recent articles that translated the latent factor structure of the CHC model to the domains of neuropsychological tests. Based on a re-analysis of 31 datasets, Jewsbury et al. (2017) provide an empirically validated overview of classifications of 47 common outcome measures, which contains 13 of the 15 outcome measures used in this article. The only exceptions are the task completion times of the OCT and SCT. These outcome measures were loaded on processing speed.

In a recent study classifying fluency tests in the CHC framework, Jewsbury and Bowden (2016) found that although strongly related to processing speed and acquired knowledge, performance on tests such as the COWAT and CFT was best described through a separate fluency factor. This factor structure was confirmed by Agelink van Rentergem et al. (2020). Following the factor structures proposed by these two studies, we specified five cognitive domains (at the level of “broad abilities” in the CHC vocabulary): processing speed, retrieval fluency, working memory, learning efficiency, and visual-spatial processing. See Figure 1B for a visualization of the mappings.

The CHC model is sometimes specified as a hierarchical model, with “general intelligence (g)” in a second-order or bifactor hierarchy that explains the covariance commonly found among the broad abilities and/or outcome measures. We will not attempt to estimate second-order or bifactor variants of the CHC model here in order to stay close to the model specifications reported by Jewsbury and Bowden (2016) and Jewsbury et al. (2017).

## Results

In cases of non-normality, test scores underwent log- or inverse transformations. Transformations were necessary for the task completion times of the TMT, Stroop, SCT and OCT (see Table 3 for descriptive statistics and significant demographic predictors).

**TABLE 3:** Means and standard deviations of relevant measures.

**Test**		**Outcome measures**	**Raw mean (SD)**	**Significant predictors**
1.	Trail-Making Test A^*a*^	Completion time (s)^*a*^	33.29(11.62)	A
2.	Trail-Making Test B^*a*^	Completion time (s)^*a*^	67.90(23.52)	A, E, S
3.	Digit span forward	Total score^*a*^	6.91(1.90)	A, E, S
		*Completion time (s)*	130.49(36.73)	
4.	Digit span backward	Total score^*a*^	6.49(1.97)	A, E
		*Completion time (s)*	113.04(37.99)	
5.	COWAT	Total correct trial 1–3^*a*^	37.48(10.31)	A, E, S
6.	CFT, category ‘animals’	Total correct^*a*^	24.65(5.61)	A, E
7.	Stroop color-naming^*b*^	Completion time (s)^*a*^	74.90(15.15)	A
8.	Stroop interference^*b*^	Completion time (s)^*a*^	127.49(31.13)	A, E, S
9.	RAVLT learning trials	Total score trial 1–5^*a*^	40.78(10.81)	A, E, S
		*Completion time (s)*	159.64(68.98)	
10.	RAVLT delayed recall	Total score^*a*^	8.56(3.27)	A, E, S
		*Completion time (s)*	41.07(19.17)	
		*Learning-Recall Time Delay*	11.17(3.29)	
11.	ROCFT copy	Total score^*a*^	32.52(2.87)	A, E
		*Completion time (s)*	149.36(53.93)	
12.	ROCFT immediate recall	Total score^*a*^	18.67(6.27)	A, S
		*Completion time (s)*	112.25(40.94)	
		*Copy-Recall time delay*	5.49(1.15)	
13.	Star-Cancellation Test^*b*^	Completion time (s)^*a*^	46.86(15.70)	A
14.	O-Cancellation Test^*b*^	Completion time (s)^*a*^	90.87(34.65)	A, S
15.	Clock Drawing Test	*Not used in analyses*	−	–
16.	Card Sorting Test	*Not used in analyses*	−	–

*A = age; E = highest education (scale 0–6; UNESCO, 1997/2006): 0 = pre-primary education; 1 = primary education or first stage of basic education; 2 = lower secondary or second stage of basic education; 3 = (upper) secondary education; 4 = post-secondary non-tertiary education; 5 = first stage of tertiary education; 6 = second stage of tertiary education. S, sex; COWAT, Controlled Oral Word Association Test; CFT, Category Fluency Test; RAVLT, Rey Auditory Verbal Learning Test; ROCFT, Rey-Osterrieth Complex Figure Test;^*a*^Outcome measures used in analyses.^*b*^Presented means are raw reaction times that were transformed to meet normality assumptions.*

Table 4 provides a complete overview of all fitted models and the corresponding fit statistics. Both initial models converged normally. In their original form, as specified in Figure 1, neither the CHC nor the neuropsychological consensus model reached conventional levels of acceptable model fit (Schermelleh-Engel et al., 2003). Modification indices for both models indicated that the inclusion of a covariance specification between OCT and SCT would improve the models. This modification index was large relative to the second-to-largest value for both models (46.29 vs. 10.77 for the CHC model and 44.12 vs. 9.84 for the neuropsychological consensus model) and significant (*p* < 0.001). In addition, the change was considered to be theoretically justifiable since the two tests are highly similar. Therefore, we decided to add the covariance parameter to both models. After applying this change, the neuropsychological consensus model showed acceptable fit in terms of SRMR (0.056, ΔSRMR = 0.012), CFI (0.965, ΔCFI = 0.041) and TLI (0.950, ΔTLI = 0.057), and good fit in terms of AIC (8350.30, ΔAIC 39.65) and RMSEA (0.048, ΔRMSEA = 0.022). The CHC model showed acceptable fit on TLI (0.960, ΔTLI = 0.064), and good fit on all other fit measures [CFI = 0.972, (ΔCFI = 0.046), AIC = 8343, (ΔAIC = 44.9), RMSEA = 0.043 (ΔRMSEA = 0.026)]. However, the chi square measures were statistically significant for both models.

**TABLE 4:** Fit statistics of all confirmatory and exploratory models.

	**χ^2^ (df)**	***p***	**SRMR**	**CFI**	**TLI**	**AIC**	**RMSEA [95% CI]**
**Neuropsychological consensus model**							
1. Initial model	140.97 (65)	<0.001	0.068	0.924	0.893	8389.95	0.070 [0.054, 0.086]
2. Covariance between OCT and SCT^*a*^	99.32 (64)	0.003	0.056	0.965	0.950	8350.30	0.048 [0.028, 0.066]
3. Executive functioning and processing speed merged^*a*^	109.49 (68)	0.001	0.057	0.958	0.944	8352.48	0.051 [0.032, 0.068]
**Cattell-Horn Carroll model**							
1. Initial model	138.92 (65)	<0.001	0.058	0.926	0.896	8387.90	0.069 [0.053, 0.085]
2. Covariance between OCT and SCT^*a*^	92.01 (64)	0.012	0.044	0.972	0.960	8343	0.043 [0.021, 0.061]

*χ^2^, chi square; df, degrees of freedom; CFI, comparative fit index; AIC, Akaike information criterion; RMSEA, root mean square error of approximation; OCT, O-Cancellation Test; SCT, Star-Cancellation Test; TMT, Trail-Making Test; CHC, Cattell-Horn-Carroll; Gr, Retrieval fluency.^*a*^Exploratory model change.*

We formally compared the model fit of the neuropsychological consensus model and the CHC model through the Vuong test for non-nested models. The test of distinguishability based on the observed data was statistically significant, ω^2^ = 0.17, *p* = 0.002. However, the non-nested likelihood ratio test indicated that neither model provided a better fit, *z* = −0.57, *p* = 0.72, and the 95% confidence interval of the AIC difference contained zero [−17.65, 32.25]. Thus, both model specifications represented the data equally well. Tables 5, 6 present the factor loadings of these models and Table 7 presents correlations between cognitive domains. The sample covariance matrix and lavaan syntax for all model versions described here are provided in the Supplementary Materials.

**TABLE 5:** Standardized factor loadings of the neuropsychological consensus model.

	**Estimate**	***SE***	**Z (*p*)**
**Executive functioning**			
Trail-Making Test B	0.64	0.06	9.97 (< 0.001)
Stroop interference	0.61	0.07	9.21 (< 0.001)
COWAT	0.44	0.07	6.32 (< 0.001)
CFT	0.46	0.06	7.18 (< 0.001)
**Processing speed**			
Trail-Making Test A	0.66	0.07	9.39 (< 0.001)
Stroop color-naming	0.58	0.07	8.29 (< 0.001)
OCT	0.45	0.07	6.35 (< 0.001)
SCT	0.34	0.07	4.63 (< 0.001)
**Working memory**			
Digit Span forward	0.65	0.08	8.31 (< 0.001)
Digit Span backward	0.74	0.08	8.95 (< 0.001)
**Memory**			
RAVLT learning trials	0.44	0.07	6.18 (< 0.001)
RAVLT delayed recall	0.43	0.07	6.00 (< 0.001)
ROCFT immediate recall	0.76	0.08	9.32 (< 0.001)
**Visual-spatial processing**			
ROCFT copy	1.26	0.06	21.86 (< 0.001)

*COWAT, Controlled Oral Word Association Test; CFT, Category Fluency Test; OCT, O-Cancellation Test; SCT, Star-Cancellation Test; RAVLT, Rey Auditory Verbal Learning Test; ROCFT, Rey-Osterrieth Complex Figure Test.*

**TABLE 6:** Standardized factor loadings of the Cattell-Horn-Carroll model.

	**Estimate**	***SE***	**Z (*p*)**
**Retrieval fluency (Gr)**			
COWAT	0.51	0.08	6.77(< 0.001)
CFT	0.51	0.07	7.09(< 0.001)
**Processing speed (Gs)**			
Trail-Making Test A	0.63	0.07	8.98(< 0.001)
Trail-Making Test B	0.67	0.07	10.08(< 0.001)
Stroop color-naming	0.55	0.07	7.98(< 0.001)
Stroop interference	0.63	0.07	9.27(< 0.001)
OCT	0.43	0.07	6.24(< 0.001)
SCT	0.33	0.07	4.62(< 0.001)
**Working memory (Gwm)**			
Digit Span forward	0.65	0.08	8.06(< 0.001)
Digit Span backward	0.74	0.09	8.62(< 0.001)
**Learning efficiency (Gl)**			
RAVLT learning trials	0.87	0.06	13.91(< 0.001)
RAVLT delayed recall	0.87	0.06	13.82(< 0.001)
**Visual-spatial processing (Gv)**			
ROCFT copy	0.39	0.07	5.87(< 0.001)
ROCFT immediate recall	0.95	0.12	7.90(< 0.001)

*Broad factor abbreviations shown between parentheses are the conventional CHC nomenclature. COWAT, Controlled Oral Word Association Test; CFT, Category Fluency Test; OCT, O-Cancellation Test; SCT, Star-Cancellation Test; RAVLT, Rey Auditory Verbal Learning Test; ROCFT, Rey-Osterrieth Complex Figure Test.*

**TABLE 7:** Correlations between cognitive domains in the neuropsychological consensus model and Cattell-Horn-Carroll model.

**Neuropsychological Consensus model**					**Cattell-Horn-Carroll Model**				
	**1.**	**2.**	**3.**	**4.**		**1.**	**2.**	**3.**	**4.**
1. Executive functioning	−				1. Retrieval fluency (Gr)	−			
2. Processing speed	0.94^*a*^	−			2. Processing speed (Gs)	0.78^*a*^	−		
3. Working memory	0.62^*a*^	0.45^*a*^	−		3. Working memory (Gwm)	0.58^*a*^	0.52^*a*^	−	
4. Memory	0.59^*a*^	0.44^*a*^	0.28^*a*^	−	4. Learning efficiency (Gl)	0.53^*a*^	0.36^*a*^	0.30^*a*^	−
5. Visual-spatial processing	0.12^*a*^	0.09	0.15^*a*^	0.37^*a*^	5. Visual-spatial processing (Gv)	0.28^*a*^	0.40^*a*^	0.18^*a*^	0.40^*a*^

*^*a*^Significant at 0.05.*

In the neuropsychological consensus model, a high correlation of 0.94 was found between executive functioning and processing speed. Since this might suggest that their indicators measured the same underlying cognitive domain, we constructed an additional model based on model 2 in which executive functioning and processing speed were merged into one more general domain. Fit indices of this exploratory model were worse than the model containing both cognitive domains (i.e., model 2). The chi square difference test was significant, χ^2^(4) = 10.18, *p* = 0.038, suggesting that the more constrained model version containing both executive functioning and processing speed as separate domains was a better fit of the data than the neuropsychological consensus model in which these domains were merged.

## Discussion

Our findings demonstrate that the set of digitized neuropsychological tests in the newly developed DCTB measure the same cognitive domains to which they are commonly associated in the literature. With only a minor change to the model specifications, both the neuropsychological consensus model and the CHC model provided an acceptable to good fit to the data, and there was no evidence to favor one model over another. In the neuropsychological consensus model there was a high correlation between executive functioning and processing speed. This correlation was likely due to the fact that both of these cognitive domains consisted of timed tasks with several outcome measures of the same test loading on both domains. Such issues are sometimes addressed by calculating ratio or difference scores, but the resulting compound scores contain the measurement errors of both individual outcome measures, making them less reliable. Despite the high correlation, merging executive functioning and processing speed into one domain reduced model fit, indicating that both cognitive domains captured unique variance in test performance.

The neuropsychological literature contains several classifications of cognitive functioning with overlapping cognitive domains and associated tests, owing to the fact that most cognitive tests are multideterminant. The neuropsychological consensus model tested here was in line with multiple CFAs in the literature. The memory and working memory domains are generally seen as separable domains in the neuropsychological literature (Dowling et al., 2010; Hayden et al., 2011; Park et al., 2012), although working memory tests are sometimes grouped with attention. Interestingly, Dowling et al. (2010); Hayden et al. (2011), and Park et al. (2012) all included a merged executive functioning/processing speed domain which was not fully supported by our data. The visual-spatial domain is also featured in these studies, but more diverse in terms of selected indicators. Finally, our use of completion times of two cancellation tests to form a processing speed domain was supported by Siedlecki et al. (2008). However, it should be noted that although these studies reported good model fit, only Dowling et al. (2010) and Hayden et al. (2011) reached the cut-off values for model fit that were used in the present study.

Specification of the CHC model was more straightforward given its derivation from a single theoretical framework. We replicated recent findings by Jewsbury et al. (2017) and Agelink van Rentergem et al. (2020), showing that the model provides an acceptable to good fit in a neuropsychological context. In addition, we replicated the finding by Jewsbury and Bowden (2016) that fluency tests share common variance with measures of processing speed (the correlation between these domains being 0.78 in our CHC model) but can be regarded as a separate retrieval fluency domain in the CHC framework.

Besides providing initial evidence of validity, our study also allowed a comparison between a traditional neuropsychological factor structure and one defined by CHC theory in the digital domain. Arguably the largest point of disagreement between the neuropsychological and CHC literature concerns the existence of a separate executive functioning domain, which is generally included in traditional neuropsychological models (Strauss et al., 2006; Lezak et al., 2012) but does not have a place in the CHC taxonomy. Tests that are traditionally considered to measure executive functioning have been found to load on several CHC factors, such as processing speed and retrieval fluency (Salthouse, 2005; Jewsbury et al., 2017). This has been taken as evidence that executive functioning as a unitary construct confounds several cognitive processes (Schneider and McGrew, 2018), or does not constitute a distinct cognitive construct (Kovacs and Conway, 2016).

The findings reported here do not warrant such strong conclusions. Despite the strong correlation between executive functioning and processing speed in the neuropsychological consensus model, these domains could be reliably distinguished. Thus, as a unitary construct in the neuropsychological model, executive functioning captures unique variance in test performance that is not related to the other cognitive domains. In general, we did not have enough evidence to favor either the neuropsychological consensus model or the CHC model. Although the fit statistics of the CHC model were slightly better, a formal significance test could not differentiate between the models. It is possible that a difference would arise in a larger sample, as the CHC model was shown to provide the best fit in a sample of 60,398 participants (Agelink van Rentergem et al., 2020). However, if extremely large sample sizes are required to clearly distinguish between the two models, the question arises whether such differences are clinically meaningful. Furthermore, a solid factor structure in a sample of healthy subjects is not necessarily evidence for a model’s clinical utility. In fact, the CHC model has been found to be lacking in this respect (Wasserman, 2019). Future research including data from cognitively impaired individuals will be required to gather data on sensitivity and specificity.

The current study has a number of limitations. First, although our study showed the factor structure of the DCTB to be in line with the mostly paper-and-pencil based factor structures in the literature, the current findings offer only initial evidence for validity of our DCTB. A full assessment of validity requires measurement invariance studies, with participants completing both a digital and paper-and-pencil version of the same test battery. Such a comparison was outside of the scope of the present study. Second, we were not able to assess the validity of the full DCTB. Performance on the CDT could not be added to the model due to a ceiling effect. In addition, visual-spatial processing in the neuropsychological consensus model was only defined by ROCFT copy, so conclusions about the structural validity of the ROCFT could only be based on the CHC model. Third, at the time of data collection, parts of the DCTB were not yet automated. Tests that required a verbal response (e.g., Stroop and COWAT) were scored manually as sophisticated speech recognition techniques were not yet implemented. Additionally, scoring of the ROCFT were done manually since the scoring algorithm is still under development. These final steps in automation could have an influence on validity.

To conclude, the current study provides initial evidence of validity for a newly developed DCTB. Through CFA, it is shown that the tests generally load on the cognitive domains that were specified based on existing literature. Interestingly, no clear preference in terms of model fit can be given to either the neuropsychological consensus model or the CHC model, suggesting that they are both viable alternatives in a neuropsychological context. Adding to a growing body of literature on the advantages of digital technology in neuropsychological practice, the current study demonstrates the potential of the DCTB in quantifying cognitive functioning.

## Data Availability Statement

All datasets generated for this study are included in the article/Supplementary Material.

## Ethics Statement

The studies involving human participants were reviewed and approved by the Internal Committee for Biomedical Experiments, Philips Research and the Medical Ethical Review Board (METC), University Medical Center Utrecht. The patients/participants provided their written informed consent to participate in this study.

## Author Contributions

SV, RD, LK, BS, and JM contributed to conception and design of the study, and to the model specifications. SV performed the analyses and wrote all drafts of the manuscript. RD, LK, BS, JM, and GE provided feedback and rewrote sections of the manuscript. All authors approved the submitted version of the manuscript.

## Conflict of Interest

SV, RD, BS, LK, and GE were employed by Philips. JM received consultation fees from Philips.
