# Supplementary material for: Evidence of Validity for a Newly Developed Digital Cognitive Test Battery
Source: Front Psychol. 2020 Apr 24;11:770. doi: 10.3389/fpsyg.2020.00770 (PMC7194127; doi:10.3389/fpsyg.2020.00770)
Supplement: Supplementary file 1 [file Data_Sheet_1.pdf]

Supplementary Material

1 Supplementary Data

Here, we provide the *lavaan* syntax for all models specified in the main text. Latent factors are specified using the "=~" operator and covariances are specified using the "~~" operator. For more information on the *lavaan* syntax, see Rosseel (2012).

1.1 Traditional neuropsychological model – Model 1

```
'
# Latent factors
EF =~ NA * tmt_b + cft + cowat + stroop_interference
M =~ NA * ravlt_learning_trials + ravlt_delayed_recall + rocft_immediate_recall
PS =~ NA * sct + oct + tmt_a + stroop_color
WM =~ NA * digitspan_forward + digitspan_backward
VS =~ NA * rocft_copy

# Covariances
stroop_color ~~ stroop_interference
tmt_a ~~ tmt_b
ravlt_learning_trials ~~ ravlt_delayed_recall

# Standardization of latent factor variances
EF ~~ 1 * EF
PS ~~ 1 * PS
WM ~~ 1 * WM
M ~~ 1 * M
VS ~~ 0.4 * VS
'
```

1.2 Traditional neuropsychological model – Model 2

```
'
# Latent factors
EF =~ NA * tmt_b + cft + cowat + stroop_interference
M =~ NA * ravlt_learning_trials + ravlt_delayed_recall + rocft_immediate_recall
PS =~ NA * sct + oct + tmt_a + stroop_color
WM =~ NA * digitspan_forward + digitspan_backward
VS =~ NA * rocft_copy

# Covariances
stroop_color ~~ stroop_interference
tmt_a ~~ tmt_b
ravlt_learning_trials ~~ ravlt_delayed_recall
sct ~~ oct
```

```
# Standardization of latent factor variances
EF ~~ 1 * EF
PS ~~ 1 * PS
WM ~~ 1 * WM
M ~~ 1 * M
VS ~~ 0.4 * VS
’
```

1.3 Traditional neuropsychological model – Model 3

```
‘
# Latent factors
EF =~ NA * tmt_a + tmt_b + cft + cowat + stroop_color + stroop_interference + sct + oct
M =~ NA * ravlt_learning_trials + ravlt_delayed_recall + rocft_immediate_recall
WM =~ NA * digitspan_forward + digitspan_backward
VS =~ NA * rocft_copy

# Covariances
stroop_color ~~ stroop_interference
tmt_a ~~ tmt_b
ravlt_learning_trials ~~ ravlt_delayed_recall
sct ~~ oct

# Standardization of latent factor variances
EF ~~ 1 * EF
WM ~~ 1 * WM
M ~~ 1 * M
VS ~~ 0.4 * VS
’
```

1.4 CHC model – Model 1

```
‘
# Latent factors
Gs =~ NA * tmt_a + tmt_b + stroop_color + stroop_interference + sct + oct
Gl =~ NA * ravlt_learning_trials + ravlt_delayed_recall
Gr =~ NA * cft + cowat
Gwm =~ NA * digitspan_forward + digitspan_backward
Gv =~ NA * rocft_copy + rocft_immediate_recall

# Covariances
stroop_color ~~ stroop_interference
tmt_a ~~ tmt_b

# Standardization of latent factor variances
Gs ~~ 1 * Gs
Gl ~~ 1 * Gl
```

```
Gr ~~ 1 * Gr
Gwm ~~ 1 * Gwm
Gv ~~ 1 * Gv
'
```

1.5 CHC model – Model 2

```
'
# Latent factors
Gs =~ NA * tmt_a + tmt_b + stroop_color + stroop_interference + sct + oct
Gl =~ NA * ravlt_learning_trials + ravlt_delayed_recall
Gr =~ NA * cft + cowat
Gwm =~ NA * digitspan_forward + digitspan_backward
Gv =~ NA * rocft_copy + rocft_immediate_recall

# Covariances
stroop_color ~~ stroop_interference
tmt_a ~~ tmt_b
sct ~~ oct

# Standardization of latent factor variances
Gs ~~ 1 * Gs
Gl ~~ 1 * Gl
Gr ~~ 1 * Gr
Gwm ~~ 1 * Gwm
Gv ~~ 1 * Gv
'
```

2 Supplementary Figures and Tables

2.1 Supplementary Tables

Table S1

Sample Covariance Matrix of Cognitive Tests Included in All Model Versions

	1.	2.	3.	4.	5.	6.	7.	8.	9.	10.	11.	12.	13.	14.	**Mean (sd)**
1. TMT A	0.959														0.019 (0.981)
2. TMT B	0.559	0.917													0.041 (0.959)
3. ST color-naming	0.379	0.340	0.965												0.038 (0.984)
4. ST Interference	0.373	0.428	0.682	0.986											0.024 (0.995)
5. COWAT	0.185	0.187	0.323	0.247	0.982										0.012 (0.993)
6. CFT	0.282	0.308	0.297	0.282	0.277	0.873									0.030 (0.936)
7. DS forward	0.195	0.224	0.242	0.272	0.299	0.164	0.981								0.012 (0.993)
8. DS backward	0.229	0.276	0.206	0.305	0.192	0.222	0.480	1.008							-0.005 (1.006)
9. OCT	0.356	0.300	0.169	0.215	0.298	0.111	0.066	0.111	0.929						0.041 (0.966)
10. SCT	0.279	0.248	0.136	0.211	0.122	0.125	0.002	0.017	0.498	0.965					0.039 (0.984)
11. RAVLT learning trials	0.214	0.208	0.209	0.168	0.262	0.233	0.136	0.207	0.135	0.059	0.978				0.001 (0.991)
12. RAVLT delayed recall	0.241	0.216	0.175	0.165	0.226	0.249	0.126	0.232	0.133	0.031	0.759	0.980			0.012 (0.992)
13. ROCFT copy	0.040	0.072	0.100	0.126	0.068	0.089	0.122	0.145	0.075	0.027	0.171	0.148	0.639		0.070 (0.801)
14. ROCFT immediate recall	0.192	0.291	0.152	0.260	0.135	0.145	0.113	0.118	0.172	0.127	0.332	0.331	0.371	0.947	0.036 (0.975)
